# Efficacy of CDK4/6 Inhibition in colorectal cancer and the role of p16 expression in predicting drug resistance

**DOI:** 10.1007/s13402-025-01080-7

**Published:** 2025-06-16

**Authors:** Julia S. Schneider, Najib Ben Khaled, Liangtao Ye, Ralf Wimmer, Linda Hammann, Alexander Weich, Christoph Suppan, Ujjwal M. Mahajan, Andreas Jung, Jörg Kumbrink, Gerald Denk, Monika Rau, Volker Kunzmann, Solveig Kuss, Jens Neuman, Julia Mayerle, Andreas Geier, Heike M. Hermanns, Enrico N. De Toni, Florian P. Reiter

**Affiliations:** 1 Department of Medicine II, Ludwig Maximilian University Munich, Munich, Germany; 2 Digestive Diseases Center, The Seventh Affiliated Hospital, Sun Yat-Sen University, Shenzhen, China; 3 Division of Clinical Pharmacology, LMU University Hospital, LMU Munich, Munich, Germany; 4 Division of Gastroenterology, Department of Medicine II, University Hospital Würzburg, Würzburg, Germany; 5 Department of Oncology, Medical University of Graz, Graz, Austria; 6 Department of Pharmacology and Toxicology, National Institute of Pharmaceutical Education and Research (NIPER), S.A.S Nagar, India; 7 Institute of Pathology, Faculty of Medicine, Ludwig Maximilian University Munich, Munich, Germany; 8 Division of Hepatology, Department of Medicine II, University Hospital Würzburg, Würzburg, Germany; 9 Division of Oncology, Department of Medicine II, University Hospital Würzburg, Würzburg, Germany

**Keywords:** CDK4/6 Inhibition, p16 expression, Colorectal cancer

## Abstract

**Introduction:**

Colorectal cancer (CRC) is a leading cause of cancer-related mortality worldwide. The use of sequential polychemotherapies has improved the survival of patients with advanced metastatic disease. However, the survival rates achieved are low, and chemotherapy-related side effects are significant. Therefore, new, efficient, and tolerable therapies are urgently needed. In this study, we investigate the efficacy of pharmacological cyclin D-dependent kinase (CDK) 4/6 inhibition and explore the relevance of p16 as predictors of susceptibility to CDK 4/6 therapy.

**Materials and methods:**

CDK 4/6 inhibitors were evaluated in native and FOLFOX- or ribociclib-resistant CRC, hepatocellular carcinoma (HCC), and breast cancer (BC) cell lines using viability, colony formation, and flow cytometry (FC)-based assays. Western blotting was employed to assess the expression of Rb and members of the INK4 family. SiRNA-based knockdown of CDK4/6 was utilized to gain insights into mechanisms of action or resistance. Tissue from 185 CRC patients was examined for the expression of p16 and its relevance for progression-free and overall survival. The prognostic relevance of cyclin-dependent kinase inhibitor 2 A (*CDKN2A*) mRNA expression data was derived from The Cancer Genome Atlas (TCGA) data.

**Results:**

Ribociclib demonstrates significant antitumoral effects in various CRC, HCC, and BC cell lines, similar to two other approved CDK4/6 inhibitors (palbociclib and abemaciclib). Ribociclib-resistant cell lines (Hep-3B, HCC-1937, and BT-549) exhibited higher p16 expression compared to ribociclib-sensitive cell lines. In ribociclib-sensitive cell lines, CDK4/6 inhibition led to G1 phase arrest, whereas resistant cells did not exhibit such effects. A similar phenotype could be observed upon dual siRNA based CDK4/6 knockdown in ribociclib-sensitive HuH-7 and ribociclib-resistant Hep-3B cell lines. All CRC cell lines tested showed sensitivity to ribociclib, including the FOLFOX-resistant SW620 cell line. Low mRNA expression of *CDKN2A* (p16) was associated with favorable prognosis in CRC patients. No prognostic significance was found for p16 protein expression in an early-stage CRC cohort (*n* = 185).

**Conclusion:**

Ribociclib demonstrates significant antitumoral effects across a large panel of cancer cell lines and chemoresistant models, especially in CRC. Resistance towards ribociclib is associated with high p16 expression, which is a negative prognostic marker for patients with CRC. Our findings underscore p16 as a promising biomarker for predicting ribociclib responsiveness and emphasize the need for further mechanistic studies and combination therapy approaches to overcome resistance in p16^high^ patients.

**Supplementary Information:**

The online version contains supplementary material available at 10.1007/s13402-025-01080-7.

## Introduction

Colorectal carcinoma (CRC) ranks as the third most common cancer globally and is a leading cause of cancer-related mortality, making it the second deadliest malignant disease worldwide [1].

Despite advancements in CRC treatment through surgical and local ablative techniques, many patients progress to advanced stages requiring palliative systemic treatment. Sequential polychemotherapies have doubled survival rates over recent decades; however, the median survival for patients with advanced metastatic CRC remains approximately 30 months [2].

Moreover, these treatments often result in significant side effects, with low objective response rates (ORR) ranging from 10 to 30% in second-line settings and less than 10% in third-line settings [3]. Hence, there is an urgent need for new, effective, and tolerable therapies for advanced CRC [3].

**Fig. 1:**
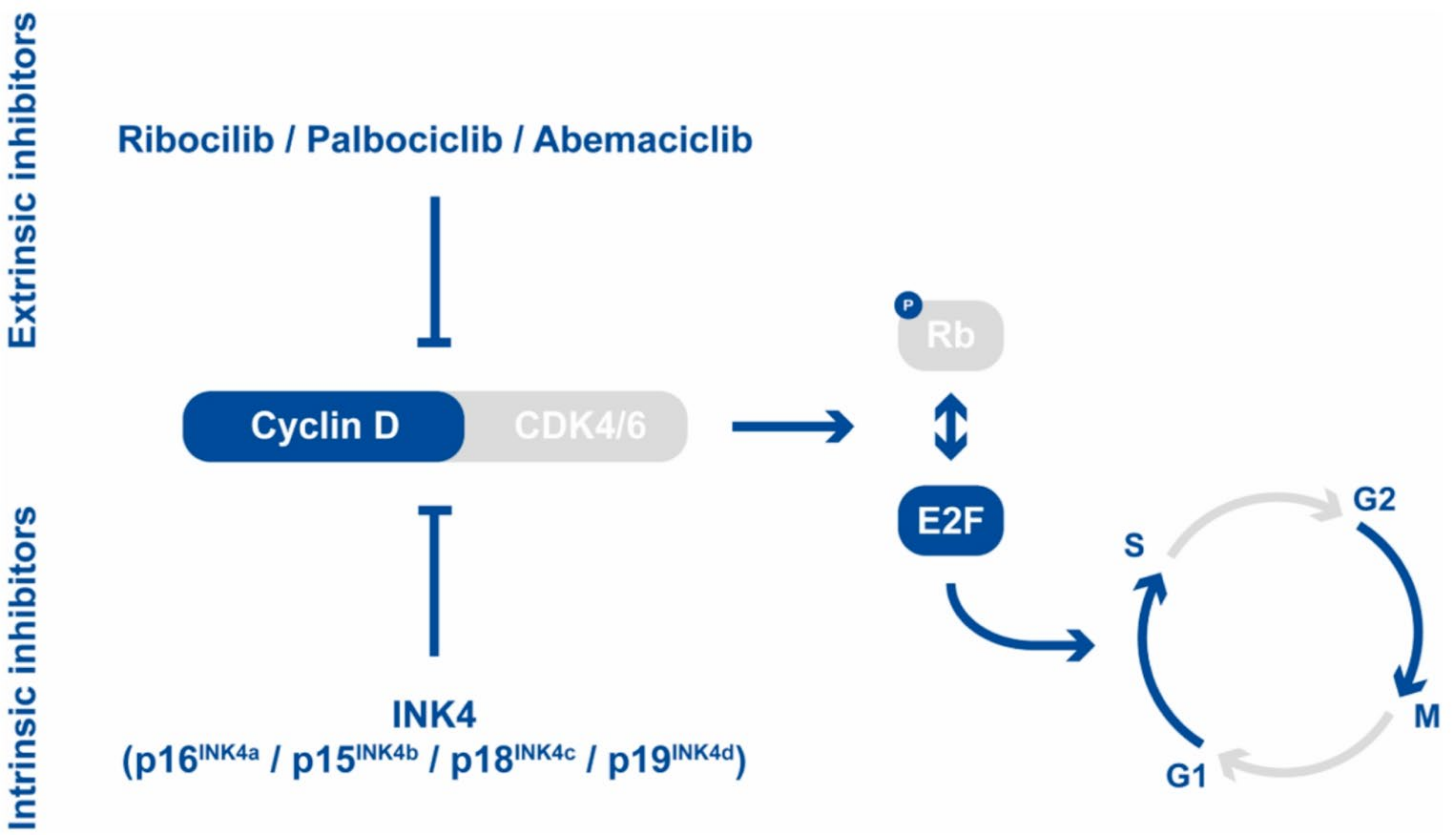
The Cyclin D - CDK4/6 - Rb signaling pathway and its endogenous and exogenous inhibition by ribociclib, palbociclib, and abemaciclib. The illustration depicts the signaling cascade of the Cyclin D - CDK4/6 - Rb pathway and its intrinsic and extrinsic inhibition (modified accordingly to Hortobagyi et al. [33])

The CDK4/6 signaling pathway plays a crucial role in regulating cell proliferation [4]. Cyclin D binds to the CDK4/6 complex, promoting the transition from G1 to S phase by phosphorylating the Retinoblastoma protein (Rb), which acts as a tumor suppressor [5]. This pathway can be inhibited by members of the INK4 family (such as p16^INK4a^, p15^INK4b^, p18^INK4c^, and p19^INK4d^) or by clinically available CDK4/6 inhibitors like ribociclib, palbociclib and abemaciclib [5, 6]. (Fig. 1).

Recent studies have shown that the ratio of Rb to its intrinsic inhibitors, particularly p16^INK4a^, can predict the effectiveness of ribociclib in hepatocellular carcinoma (HCC) [7]. In these studies, ribociclib-resistant Hep-3B cells exhibited high p16^INK4a^ expression and low Rb protein levels [7]. Preclinical research across different tumor types has consistently linked low Rb levels or functional loss of Rb to resistance against CDK4/6 inhibitors [8–10].

Interestingly, in this regard, the comprehensive study by Palafox et al. validated that resistance to CDK4/6 inhibitors in breast cancer (BC) can be associated with heterozygous Rb loss and high p16 expression, which was therefore validated as prognostic marker for resistance [11].

Despite these promising initial findings, validated biomarkers for resistance to CDK 4/6 inhibitors are still lacking in clinical practice. This is particularly concerning, as approximately 10% of BC patients treated with CDK 4/6 inhibitors either exhibit de novo resistance or develop acquired resistance during therapy [12]. Consequently, there is a high need for reliable biomarkers to predict resistance upfront and for effective strategies to overcome it.

The present study aims to evaluate the antitumor effects of inhibiting the cyclin D-CDK4/6 pathway using CDK4/6 inhibitors in a broader range of tumor entities with a special focus on CRC. CDK4/6 inhibitors, such as ribociclib, palbociclib and abemaciclib, are currently approved for treating hormone receptor-positive, HER2-negative BC. These inhibitors have demonstrated improved PFS when used in combination with endocrine therapy [13, 14]. CDK4/6 inhibitors are generally well-tolerated, with myelosuppression being a common manageable side effect in clinical practice [15]. Consequently, there is potential for its use in late-line settings or in combination with polychemotherapies. We hypothesize that the balance between Rb and its INK4 family inhibitors, such as p16, can determine the relevance of the cyclin D-CDK4/6 pathway in tumor survival and its susceptibility to CDK4/6 inhibitors.

## Materials and methods

### Cell lines, culture conditions and chemicals

In this study, human cancer cell lines from three different tumor entities were investigated: BC with MDA-MB-231, MCF-7, HCC-1937, and BT-549 cells; HCC with HuH-7 and Hep-3B cells; and CRC with SW-48, HT-29, HCT-116, DLD-1, SW-620, Colo-205, and SNU-C2A cells (suppl. Table 1). Cells were cultured in a humidified atmosphere with 5% CO2 and 21% O2 at 37 °C. All cell lines used in this study underwent genetic fingerprinting analysis by an external independent institution (Leibniz-Institut; DSMZ-Deutsche Sammlung von Mikroorganismen und Zellkulturen GmbH) to confirm authenticity and exclude contamination. Ribociclib, palbociclib, abemaciclib and 5-FU were purchased from Selleckchem (USA). Oxaliplatin was purchased from medac GmbH (Germany). FOLFOX-resistant cells were generated from native SW-620 cells by continuous incubation with increasing concentrations of 5-FU and oxaliplatin over at least 6 months, resulting in a final FOLFOX concentration of 5 µM 5-FU and 125 nM oxaliplatin (accordingly to [16]).

Ribociclib-resistant cells were developed from original HCT-116 cells through sustained exposure to escalating concentrations of ribociclib over 6 months, culminating in a final ribociclib concentration of 12.8 µM.

### 3D culture

DLD1 cells were seeded in a 3D culture containing 20 µL Matrigel/BME2 (Pathclear) and were subsequently stained with Hoechst and PI at a final concentration of 1% (v/v) each.

### Cell viability assay

For assessment of cell viability, SYBR Green assays (Lonza, Germany) were performed. Densitometric values were expressed as values normalized to the controls, as previously described [17].

### Flow cytometry

Cells were harvested from 6-well plates. After propidium iodide staining (Sigma, Germany), flow cytometry (FC Accuri C6 flow cytometer-BD Biosciences, San Jose, CA USA and BD LSRFortessa Cell Analyzer, BD Biosciences, San Jose, CA, USA) was performed. Apoptosis was quantified by calculating the fraction of cells with a sub diploid DNA content (sub G1). FlowJo™ Software for Windows, Version 10. Ashland, OR: Becton, Dickinson and Company; 2023 was used for data analysis.

### Colony formation assay

Cells were seeded in 6-well plates and incubated with ribociclib for 2 weeks. Next, colony formation was evaluated after fixation in 10% paraformaldehyde and staining with crystal violet for 15 min (Sigma Aldrich, Germany).

### Western blotting

Proteins were loaded in equal amounts, separated by SDS-PAGE and transferred to PVDF membranes (Millipore, Germany). The membranes were incubated with monoclonal antibodies directed against Rb (Cell Signaling Technology, USA, #9309), pRb (Cell Signaling Technology, USA, #3590), p16 (Abcam, UK, ab108349), Cyclin D1 (Cell Signaling Technology, USA, #2922), CDK4 (Cell Signaling Technology, USA, #12790), CKD6 (Cell Signaling Technology, USA, #13331) and GAPDH (Abcam, UK, ab8245) which was used as the loading control, followed by HRP-conjugated anti-mouse (BIO-RAD, USA, 1706516) or anti-rabbit antibodies (BIO-RAD, USA, 1706515).Immunoreactions were visualized using a SuperSignal West Dura and Femto (Thermo Fischer Scientific, USA) and detected with a ChemoCam (INTAS, Germany). All antibodies were diluted as recommended.

### SiRNA-mediated gene silencing

Transient siRNA-based knockdowns were employed for mechanistic analysis, utilizing dual knockdown of CDK4 and CDK6 (Dharmacon, USA). Oligofectamine (Thermo Fischer Scientific, USA) was used to initiate the knockdown. The efficacy of all knockdowns was validated by western blotting.

### Synergism analysis

To investigate potential synergism, Colo205 cells were treated with ribociclib, FOLFOX or a combination of both using different dosages up to eight times the previously determined IC50. Synergism analyses were performed according to the method proposed by Chou [18].

### Tissue microarray analysis

A tissue microarray comprising 185 patients with UICC stage CRC was analysed for p16 expression. The prognostic role of p16 overexpression, defined as expression levels above the median, was investigated in terms of progression-free survival and overall survival. The samples were analyzed using a monoclonal antibody against p16 (Medac, Germany, monoclonal mouse, clone p16 Ink4a G175-405), Ventana Benchmark Ultra and Ventana OptiView.

### The cancer genome atlas data analysis

Open-access RNA sequencing data for CRC samples (*n* = 286) were retrieved from the Cancer Genome Atlas (http://cancergenome.nih.gov/) and analysed using UALCAN (http://ualcan.path.uab.edu/analysis.html (accessed on 25 May 2024) [19, 20]. For survival analysis, patients were categorized based on *CDKN2A* (encoding p16) RNA expression using the median FPKM value as the cutoff.

### Human samples

The use of all human material presented in this article was approved by the ethics committee of the Faculty of Medicine of the University of Munich (Project ID: 20–656).

### Statistical analysis

Statistical calculations were performed with GraphPad Prism (GraphPad Software, USA) or SPSS 25 software package (IBM, USA) using analysis of variance (ANOVA), Kruskal-Wallis test, Mann-Whitney U tests or t-tests. P values < 0.05 were considered statistically significant. Data are presented as the means ± standard errors of the mean.

### Graphics

GraphPad Prism (GraphPad Software, USA), Adobe Illustrator CC 2019 (Adobe, USA) and Adobe Photoshop CC 2019 were used to generate the figures and graphics.

## Results

### CDK4/6 Inhibition exerts its antitumoral effects depending on the expression of p16^INK4A^

In this study, we investigated the antitumoral effects of a CDK4/6 inhibition in CRC.

**Fig. 2:**
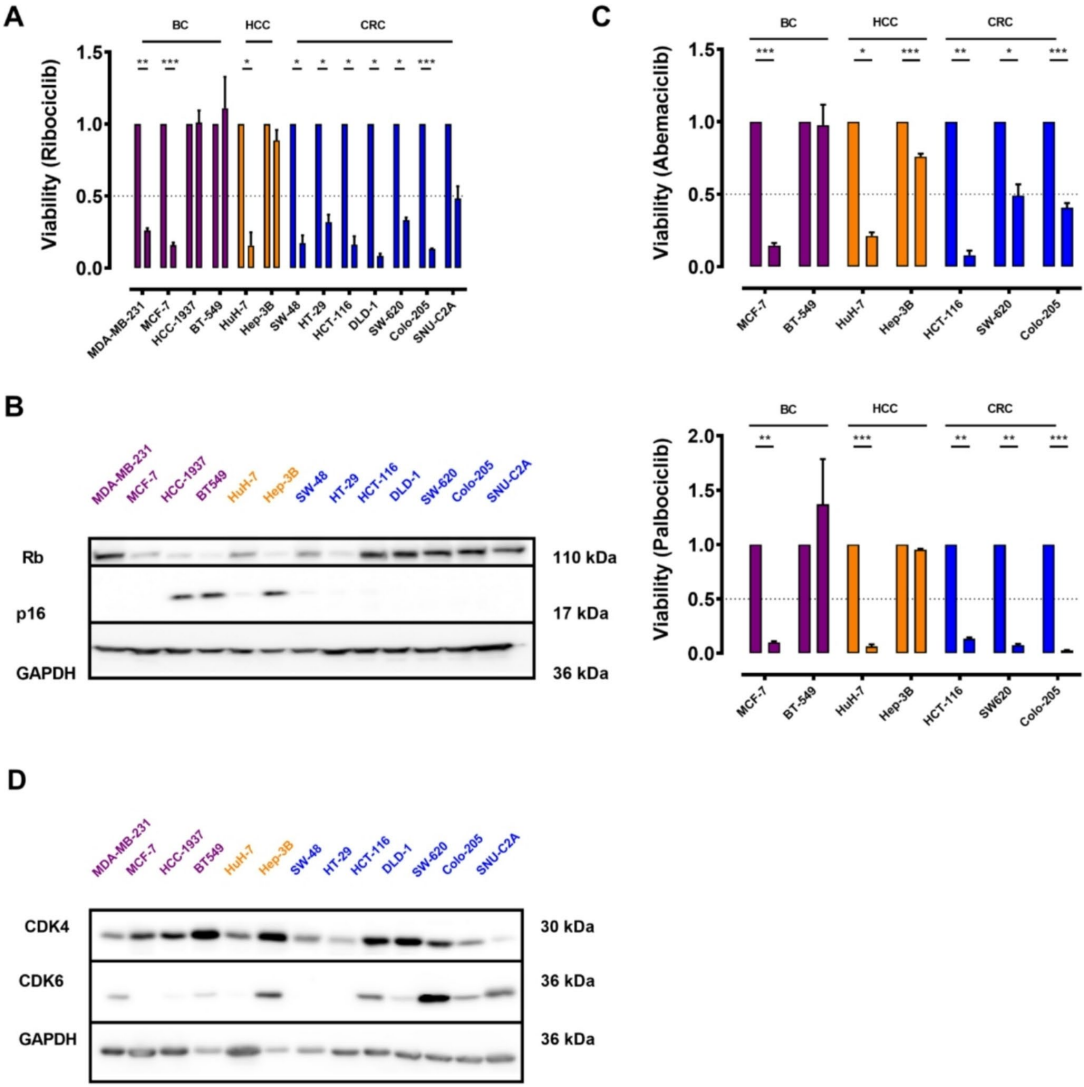
The antiproliferative effect of three approved CDK4/6 inhibitors correlates with p16 expression in various tumor entities. (**A**) BC cell lines (purple bars), HCC cell lines (yellow bars), and CRC cell lines (blue bars) were treated with 3000 nM Ribociclib for 6 days. Cell viability was determined by SYBR green assay (*n* = 3; * *p* < 0.05, ** *p* < 0.01, *** < 0.001; t-test). (**B**) Rb and p16 protein expression in the indicated cell lines determined from whole cellular extracts using SDS-PAGE/Western blot. Representative blots of 3 independent experiments are shown. (**C**) The antiproliferative effects of additional CDK4/6 inhibitors (abemaciclib 111 nM and palbociclib 1000 nM) are shown as described for (**A**) (*n* = 3; * *p* < 0.05, ** *p* < 0.01, *** *p* < 0.001; t-test). (**D**) CDK4 and CDK6 protein expression in the indicated cell lines determined from whole cellular extracts using SDS-PAGE/Western blot. Representative blots of 3 independent experiments are shown

**Fig. 3:**
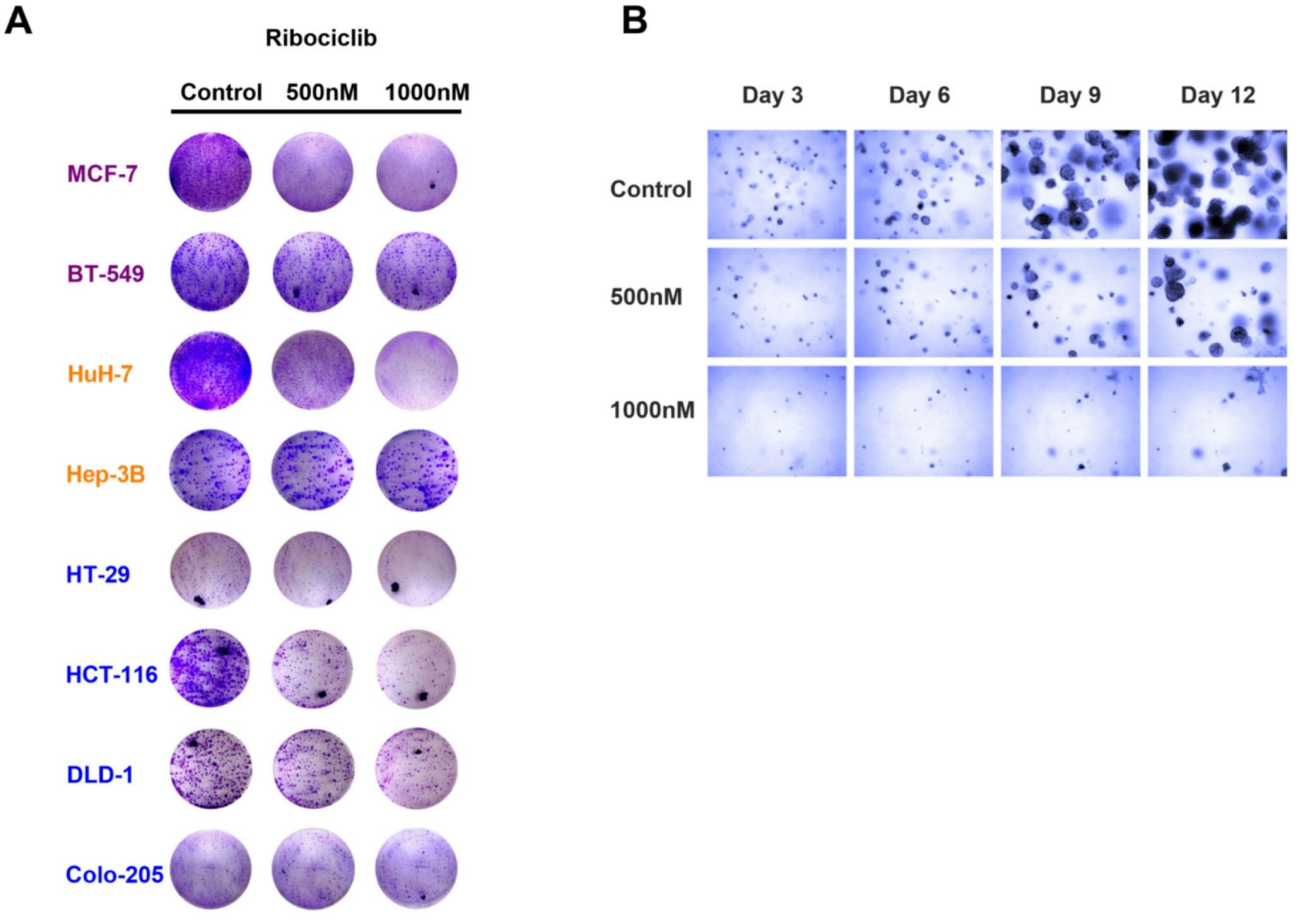
The antiproliferative effect of Ribociclib in different tumor cell entities. (**A**) The figure shows representative images of colony formation assays (*n* = 3–4). (**B**) The presented Matrigel culture illustrates the antiproliferative effects of Ribociclib in a representative image of a CRC cell line (DLD-1) (*n*)

The cell lines were evaluated with respect to their basal expression of CDK4 and CDK6 protein expression (Fig. 2). We found that nearly all CRC cell lines examined (SW-48, HT-29, HCT-116, DLD-1, SW-620, Colo-205) showed a significant response to ribociclib treatment, resulting in a marked reduction in cell viability (Fig. 2A). As a control, we evaluated the efficacy of ribociclib in various HCC and BC cell lines. Hep-3B cells were resistant, while HuH-7 cells were highly susceptible to ribociclib treatment (Fig. 2A), consistent with previous findings from our group [7]. In the BC cell lines used in this study, MDA-MB-231 and MCF-7 were highly sensitive to ribociclib treatment, whereas HCC-1937 and BT-549 were resistant (Fig. 2A). Interestingly, all resistant cell lines exhibited a high p16 protein expression (Fig. 2B), while there was no correlation between resistance and CDK4/6 protein expression in the investigated cell lines (Fig. 2D). The antitumor effects of ribociclib were validated not only in colony formation assays (Fig. 3), but also in 3D culture experiments, demonstrating profound growth inhibition up to 12 days with 500 nM and 1000 nM ribociclib compared to the control (Fig. 3). To confirm that these effects were not specific to ribociclib, we employed two additional approved CDK4/6 inhibitors, palbociclib and abemaciclib.

Here, we confirmed resistance in p16^high^ cells, such as BT-549, to abemaciclib and palbociclib, and in Hep-3B cells to palbociclib (Fig. 2C). In Hep-3B cells, a statistically significant, but numerically modest, reduction in viability was observed under abemaciclib. Thus, these effects in p16^low^ cells appear to be a drug class-specific characteristic.

**Fig. 4:**
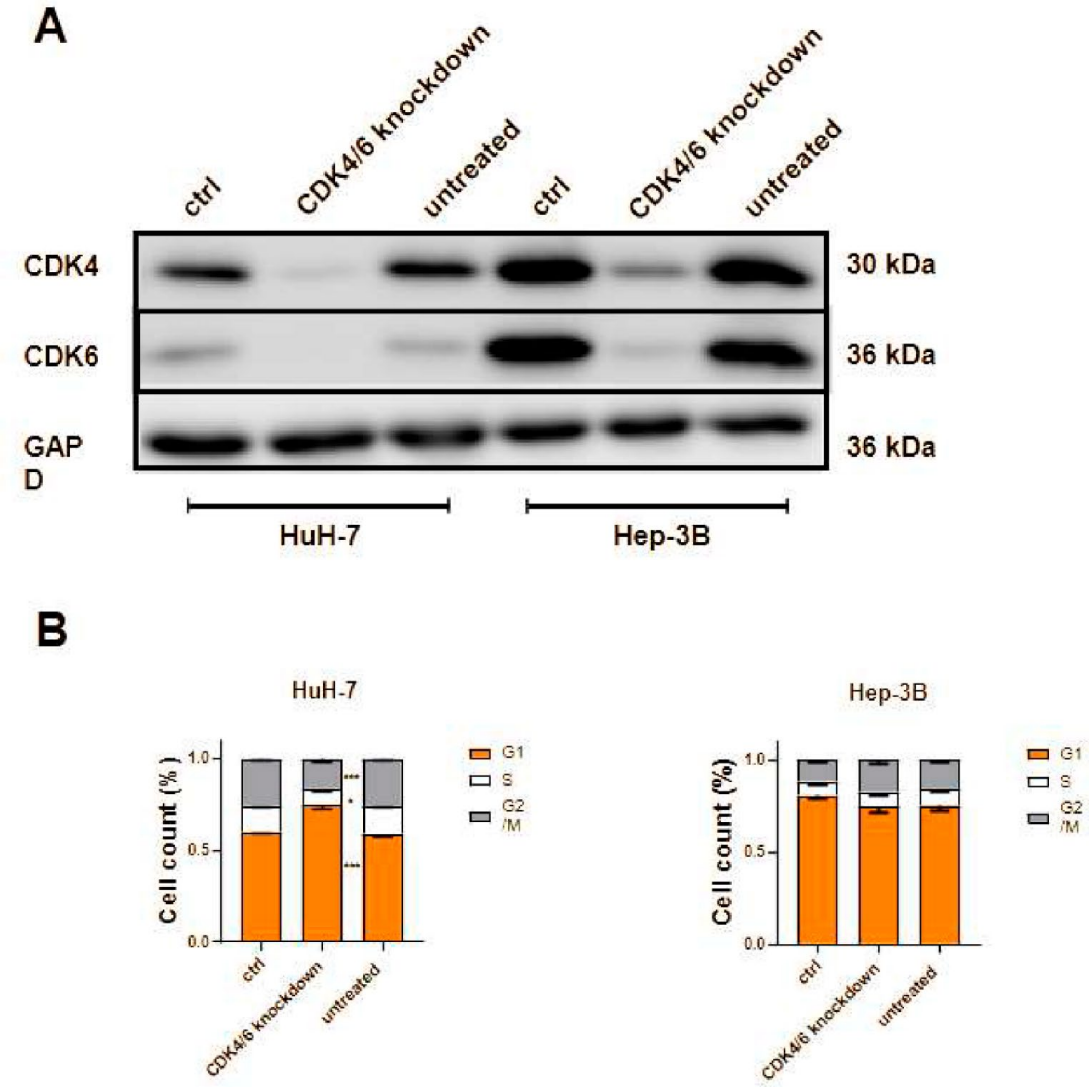
FC analysis following siRNA-based CDK4/6 knockdown. (**A**) The Western blot illustrates the efficiency of the siRNA-based knockdown in ctrl siRNA-transfected (ctrl), siRNA-transfected (CDK4/6 knockdown), and untreated (untreated) HuH-7 (left) and Hep-3B (right) cells. One representative Western blot of 3 independent experiments is shown to validate the quality of the knockdown achieved in each individual experiment. (**B**) The figure shows a FC-based analysis of cell cycle distribution after CDK4/6 knockdown in ribociclib-sensitive (HuH-7) and ribociclib-resistant (Hep-3B) cells (*n* = 3, * *p* < 0.05, ** *p* < 0.01, *** *p* < 0.001, ANOVA)

In a further set of experiments, we performed siRNA-based dual CDK4/6 knockdown in sensitive p16^low^ HuH-7 and resistant p16^high^ Hep-3B cells. Following sufficient knockdown as validated by western blotting, we observed a significant G1 arrest only in p16^low^ HuH-7 cells, while p16^high^ Hep-3B cells were unaffected by CDK4/6 knockdown (Fig. 4).

Therefore, we confirmed our findings not only by employing various pharmaceutical CDK4/6 inhibitors but also mechanistically through siRNA-based CDK4/6 knockdown. Our results demonstrate that the antitumor effects are observable across different tumor types and appear to be more correlated with p16 expression than with tissue of origin. In conclusion, our study reveals that cells with high p16 expression are resistant to CDK4/6 inhibition, suggesting that p16 expression may serve as a diagnostic biomarker to predict the efficacy of CDK4/6 inhibition in tumors.

### CDK 4/6 induces G1 arrest in p16^low^ tumor cells

**Fig. 5:**
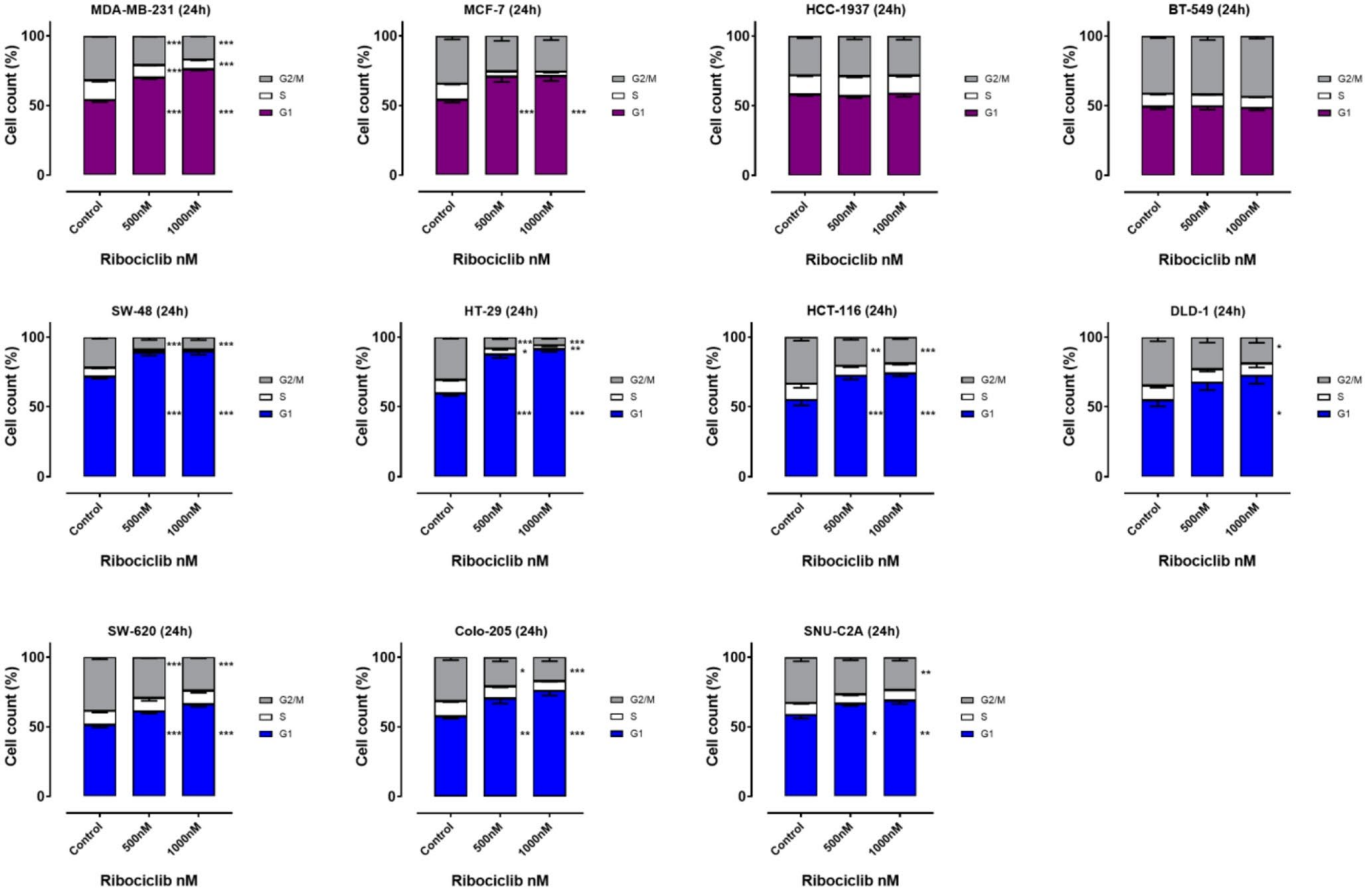
FC-based cell cycle analysis under ribociclib treatment. The indicated cell lines were cultured in the absence or presence of 500–1000 nM Ribociclib for 24 h. After propidium iodide staining DNA content of the cells was analyzes using flow cytometry (*n* = 3, * *p* < 0.05, ** *p* < 0.01, *** *p* < 0.001, ANOVA)

Consistent with these findings, all sensitive CRC and BC cell lines exhibited G1 arrest, whereas resistant HCC-1937 and BT-549 cells did not (Fig. 5). None of the cell lines investigated showed apoptosis, as indicated by analysis of the sub-G1 fraction, suggesting that the effects are due to cell cycle arrest rather than proapoptotic mechanisms (suppl. Figure 1). Thus, we conclude that CDK4/6 inhibitors exert their antitumor effects through G1 arrest in sensitive tumor cell lines, as anticipated.

### p16 expression serves as an indicator of intrinsic resistance rather than a target for overcoming resistance to ribociclib

**Fig. 6:**
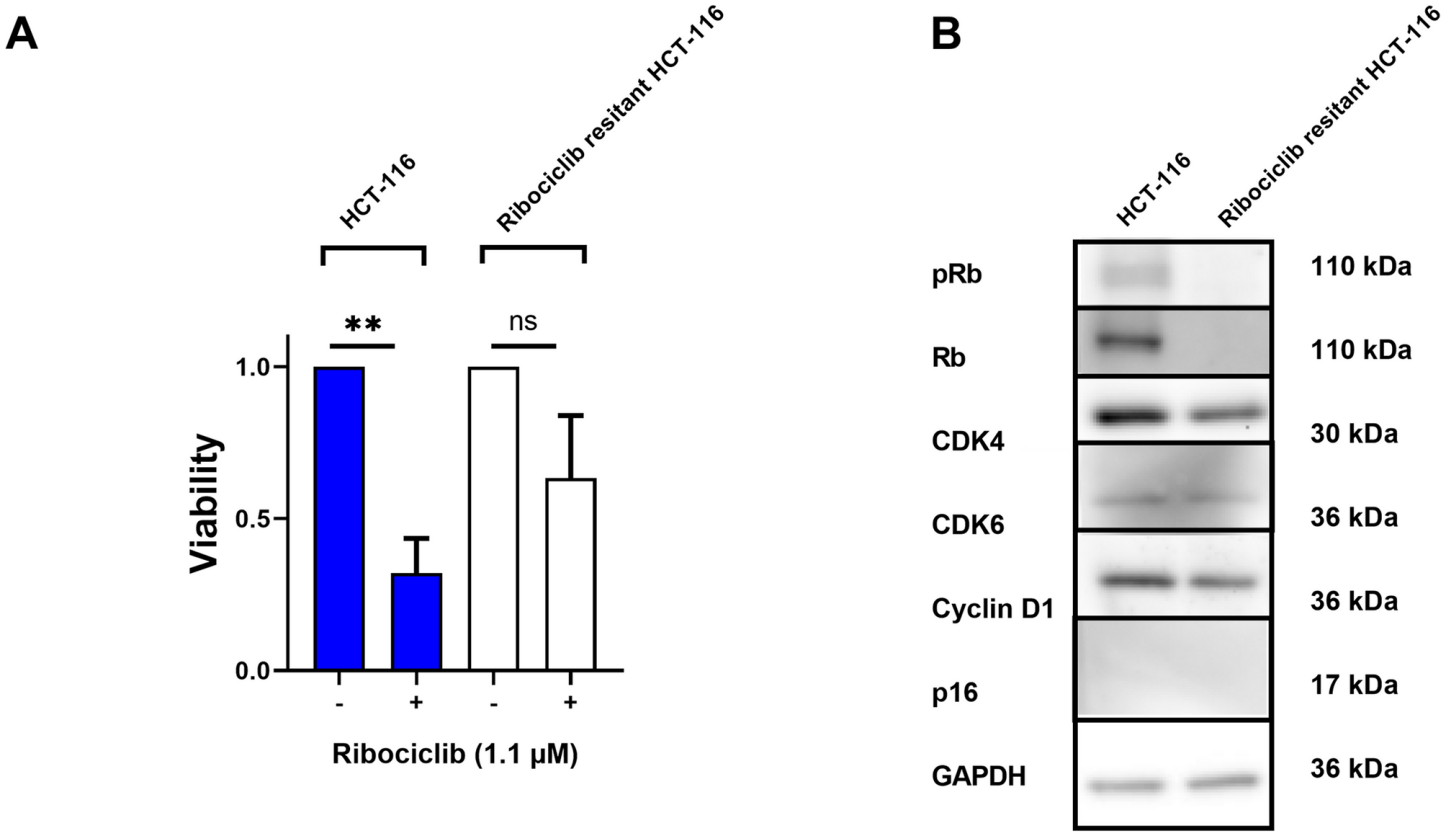
Viability and protein expression profiling of a ribociclib-sensitive and ribociclib-resistant HCT-116 cell line. (**A**) The proliferation assay illustrates the induced resistance to 1.1 µM ribociclib (*n* = 3; ** *p* < 0.01; t-test). (**B**) The Western blot shows the protein expression profile of the sensitive and resistant HCT116 cell line. Representative blots of at least 3 independent experiments are shown

We observed a p16^high^ phenotype in resistant cell lines. Based on this finding, we hypothesized that continuous ribociclib stimulation leading to ribociclib resistance could induce an extrinsic resistance mechanism, potentially involving p16 overexpression. Therefore, we established a ribociclib-resistant CRC cell line (resHCT-116), as demonstrated by the strongly increased survival of cells in the presence of 1.1 µM ribociclib (Fig. 6A). Investigation of the protein expression profile using western blotting revealed a pronounced reduction in Rb and consequently phospho-Rb expression in ribociclib-resistant HCT-116 cells, while expression level of CDK4, CDK6, Cyclin D1 and p16 remained unchanged (Fig. 6B).

In summary, we conclude that there appears to be a distinction between genuine (intrinsic) resistance to CDK4/6 inhibition, which is associated with p16^high^ expression, and introduced (extrinsic) resistance to ribociclib.

### Ribociclib does not exert synergistic antitumor effects in combination with FOLFOX but demonstrates activity in FOLFOX-resistant SW620 cells

We demonstrate that CDK4/6 inhibition has antitumor effects in CRC lines (Fig. 2A).

The optimal use of ribociclib in the treatment of CRC is currently unknown, including its potential integration into current standard therapies.

Therefore, we investigated whether ribociclib has antitumor activity in FOLFOX-resistant CRC cells or in combination with FOLFOX, which is currently used as a standard therapy for patients with advanced stage colorectal cancer. Initially, we confirmed numerically sensitivity to FOLFOX treatment across all CRC cell lines used in this study (SW-48, HT-29, HCT-116, DLD-1, SW-620, Colo-205, SNU-C2A) using a dosage of 5µM 5-FU + 125nM Oxaliplatin (suppl. Figure 2) as previously described [16]. As next step, we generated a FOLFOX-resistant CRC cell line by continuous exposure of SW-620 to FOLFOX. Cell viability assays (SYBR Green assays) of resistant SW620 and wildtype SW620 were performed to verify resistance. Subsequently, we found that ribociclib remained effective in this resistant cell line, suggesting the potential use of the inhibitor in second-line settings after the loss of efficacy of first-line FOLFOX therapy (suppl. Figure 3). However, we did not observe synergistic effects when ribociclib was combined with FOLFOX suppl. Figure 4). Therefore, we conclude that ribociclib may be ideally investigated following the loss of efficacy of first-line FOLFOX therapy in second-line treatment for CRC.

### p16 as a prognostic factor in CRC

**Fig. 7:**
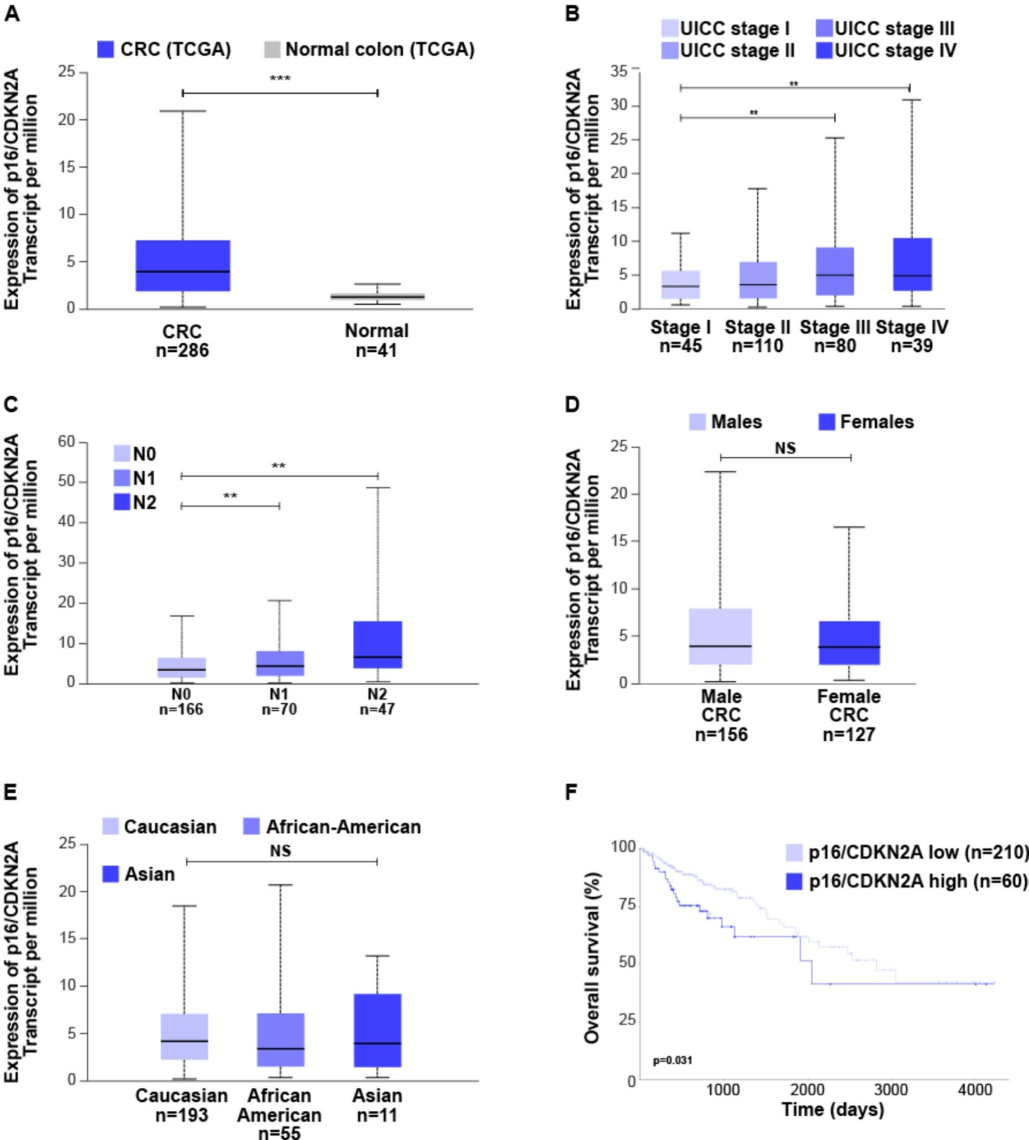
Analysis of *CDKN2A* expression in colon carcinoma (**A**) Expression of *CDKN2A*/p16 transcripts in tumor tissue compared to normal tissue. (**B**), (**C**) Expression of *CDKN2A*/p16 transcripts in different tumor stages and nodal involvement. Higher UICC-Stage as well as greater nodal involvement is associated with higher p16 expression. (**D**) *CDKN2A*/p16 transcript expression in male and female patients or (**E**) among different ethnicity. (**F**) Correlation of high *CDKN2A*/p16 transcript expression and low overall survival. RNAseq data from TCGA datasets were analyzed using UALCAN

Currently, it remains unclear which prognostic subgroup of CRC patients could benefit from a therapy with CDK4/6 inhibitors. In this study, we analyzed a large cohort of patients with UICC stage II CRC and stained their samples for p16 expression. By defining subgroups based on p16 expression (p16^high^ and p16^low^, using the median as the threshold), we did not observe differences in overall survival (mOS) (suppl. Figure 5) or progression-free survival (PFS) (data not shown) among these rather early-stage CRC patients. In order to get a better impression even of later disease stages, we took advantage of the TCGA datasets. Indeed, a highly significant enrichment of p16 transcripts (*CDKN2A*) was detectable in patients with CRC compared to normal tissue (Fig. 7A). Stratification of the patients with respect to the UICC stage confirmed no significant differences in stage I and stage II patient, while stage III and stage IV tumors expressed significantly increased *CDKN2A* mRNA (Fig. 7B). Also, a significant increase in expression could be detected in presence of nodal involvement (Fig. 7C). No significant differences were observed between male and female patients (Fig. 7D) or with respect to ethnicity (Fig. 7E). We found that high mRNA expression of p16 is significantly correlated with an unfavorable prognosis (Fig. 7F).

## Discussion

In our study, we found antitumor effects of the CDK4/6 inhibitor ribociclib in several native, but also in a FOLFOX-resistant CRC cell line. This could set the basis for further investigation of CDK4/6 inhibitors for the treatment of CRC, a malignancy where optimization of systemic therapy is urgently needed.

In the present study, we found a conspicuous association between p16 expression and reduced sensitivity to CDK4/6 inhibition by three different pharmaceutical CDK4/6 inhibitors. From a mechanistic perspective, we identified this observation as a class effect. Furthermore, our study demonstrated that CDK4/6 inhibition, performed through siRNA-based dual knockdown of CDK4 and CDK6, was ineffective in the p16^high^ Hep3B cell line.

While we mechanistically demonstrated effective inhibition of cell proliferation through siRNA-mediated dual knockdown of CDK4/6 in p16^low^ HuH-7 cells, future experiments in other p16^low^ expressing cell lines of diverse origins may help to confirm and generalize this effect. Notably, consistent with the effects of pharmaceutical CDK4/6 inhibition, our findings suggest that the observed responses are independent of tissue origin and are consistent with results from a comprehensive analysis performed by Palafox et al., who demonstrated in BC that resistance to CDK4/6 inhibitors is associated with heterozygous Rb loss and high p16 expression [11].

Thereby, our study increases the body of evidence suggesting that p16 expression could serve as a biomarker of CDK4/6 resistance not only in BC but also in other tumors as demonstrated for HCC [7].

With respect to the investigated CRC cell lines, we unfortunately did not identify a p16-expressing line to specifically validate this finding for CRC. The lack of a p16^high^ CRC cell line in our analysis reflects a limitation of this study. However, given the overlapping results regarding the effect of CDK4/6 inhibition in BC and HCC cell lines investigated here, we postulate a tumor-agnostic relevance of our findings. In the case of identifying a p16^high^ CRC line, we encourage validating our findings through future research.

The association of p16 with resistance to CDK4/6 inhibition raises the question of whether targeting p16 itself would be useful to overcome an established CDK4/6 inhibitor resistance.

Given that approximately 10% of BC patients exhibit intrinsic resistance to CDK4/6 inhibitors and that acquired resistance often develops over the course of therapy (typically after 24–48 months in first-line treatment [12]), identifying strategies to overcome these resistance mechanisms has become a critical area of research.

Promising results have emerged from targeting the PI3K/AKT/mTOR and FGFR pathways [12]. Other notable approaches include combined inhibition of CDK2 and CDK4/6, suppression of the MAPK pathway, and the use of immune checkpoint inhibitors in immunotherapy [12]. A further intriguing strategy is the combination of RAF/MEK/ERK-targeted therapies with CDK4/6 inhibitors, which has shown promising preclinical results in melanoma ([21]).

Of note, in our generated ribociclib-resistant HCT-116 cells a clear reduction in expression levels of Rb was evident. However, we do not conclude that this could serve as prognostic indicator of resistance, as we observed the same effect already under short treatment in a previous study [7].

Given the promising efficacy of CDK4/6 inhibition in reducing cell growth across a broad range of tumor types, we performed further experiments to simulate potential clinical scenarios to investigate where CDK4/6 inhibition could find its place in systemic therapy of CRC. Thus, we investigated its efficacy in combination with FOLFOX as a standard first-line therapy and in the context of FOLFOX resistance. Although there is evidence that combined CDK4/6 inhibition with oxaliplatin can mitigate resistance to neoadjuvant therapy in rectal cancer [22], and that CDK inhibitors can potentiate the efficacy of 5-FU therapy in preclinical CRC models [23], we did not find synergistic effects between ribociclib and FOLFOX in our study. However, it is important to note that we investigated a highly FOLFOX-sensitive Colo-205 cell line, which responded strongly to FOLFOX therapy. Therefore, we cannot rule out the possibility that this approach lacks the granularity needed to demonstrate synergistic effects in FOLFOX-resistant or less sensitive tumors. In light of the two mentioned studies and further work that demonstrated synergistic effects of EGFR, and/or MEK, and CDK4/6 inhibition [24–26] there is a place for CDK4/6 inhibitors to be investigated further in combination with approved CRC therapeutics.

CDK4/6 inhibition may offer therapeutic efficacy for KRAS-mutated tumors, a mutation found in up to 40% of metastatic CRC cases [27]. This mutation is also associated with resistance to anti-EGFR therapy, a key treatment strategy in advanced CRC [28]. Preclinical evidence from KRAS-induced lung cancer models highlights the critical role of CDK4 activity in tumor progression [29].

In the JUNIPER Phase III multicenter, randomized, open-label trial, abemaciclib was evaluated against erlotinib in patients with stage IV non-small cell lung cancer (NSCLC) and detectable mutations in codons 12 or 13 of the KRAS oncogene. This trial demonstrated improvements in PFS and response rates [30]. Although the primary endpoint of improved OS was not achieved, these findings provide a strong rationale for investigating CDK4/6 inhibition in CRC, particularly in KRAS-mutated subpopulations, by demonstrating antitumoral activity.

Given these results, further investigation into CDK4/6 inhibition in CRC, with an emphasis on KRAS mutations, appears crucial.

Furthermore, the limited side effects of CDK4/6 inhibition strengthen further investigation with these agents. We believe that a combination therapy would be manageable with respect to side effects from our clinical perspective. Our study demonstrated that the investigated CRC cell lines are and remained sensitive to therapy even after acquiring FOLFOX resistance. We postulate that this finding could provide a rationale for introducing CDK4/6 inhibition after FOLFOX induction in p16^low^ tumors. This approach is inspired by results from pancreatic cancer, where *BRCA*-mutated tumors are now treated with olaparib maintenance to provide chemotherapy-free time for patients after responding to platinum-based therapy [31]. This proven approach of systemic therapy could serve as a role model to include CDK4/6 inhibitors in the systemic therapy of CRC through a biomarker-driven approach.

Regarding the results from investigations of human material, we did not find prognostic relevance of p16 expression in early stage (UICC II) CRC patients. However, in investigations from the TCGA atlas, we found that in a cohort including advanced stages, there is a prognostic relevance of p16 on survival in CRC patients. Therefore, we conclude that p16^low^ patients, who showed a favorable prognosis and might be candidates for CDK4/6 inhibition, represent a prognostic subgroup that could benefit from CDK4/6 inhibition therapy for maintenance treatment.

Thus, in an ideal scenario, CDK4/6-based therapy could potentially offer chemotherapy-free intervals for CRC patients. Further studies are necessary to validate our assumptions. Also, a potential Co-inhibition of the RAS-MEK-ERK signaling pathway should be addressed in future studies [32].

Taken together, our study shows that CDK4/6 inhibition is effective in investigated CRC cell lines, independent of the presence of FOLFOX resistance. We confirm an association of high p16 expression with resistance to CDK4/6 inhibition, both pharmacologically and through siRNA methods. These observations were independent of the tissue origin of tumors, potentially indicating diagnostic validity for predicting the efficacy of CDK4/6 inhibition. Lastly, we identify a prognostically favorable patient cohort who could become candidates for maintenance therapy or after acquisition of FOLFOX resistance. Further studies are warranted to investigate the potency of CDK4/6 inhibitors for the treatment of CRC.

## Electronic supplementary material

Below is the link to the electronic supplementary material.

Supplementary Material 1

## Data Availability

Open-access RNA sequencing data for CRC samples (*n* = 286) were retrieved from the Cancer Genome Atlas (http://cancergenome.nih.gov/) and analysed using UALCAN (http://ualcan.path.uab.edu/analysis.html (accessed on 25 May 2024).
