# Supplementary material for: Efficacy of CDK4/6 Inhibition in colorectal cancer and the role of p16 expression in predicting drug resistance
Source: Cell Oncol (Dordr). 2025 Jun 16;48(5):1363–75. doi: 10.1007/s13402-025-01080-7 (PMC12528337; doi:10.1007/s13402-025-01080-7)
Supplement: Supplementary file 1 — Supplementary Material 1 [file 13402_2025_1080_MOESM1_ESM.docx]

**Supplementary material**

| **Tumor Entity** | **Cell Line** |
| --- | --- |
| **Hepatocellular carcinoma** | HuH-7 |
|  | Hep-3B |
| **Breast cancer** | MDA-MB-231 |
|  | MCF-7 |
|  | HCC-1937 |
|  | BT-549 |
| **Colorectal cancer** | SW-48 |
|  | HT-29 |
|  | HCT-116 |
|  | DLD-1 |
|  | SW-620 |
|  | Colo-205  SNU-C2A |

**Suppl. table 1 Overview of Cell Lines.** The table illustrates the cell lines used, categorized by their tumor entities of origin


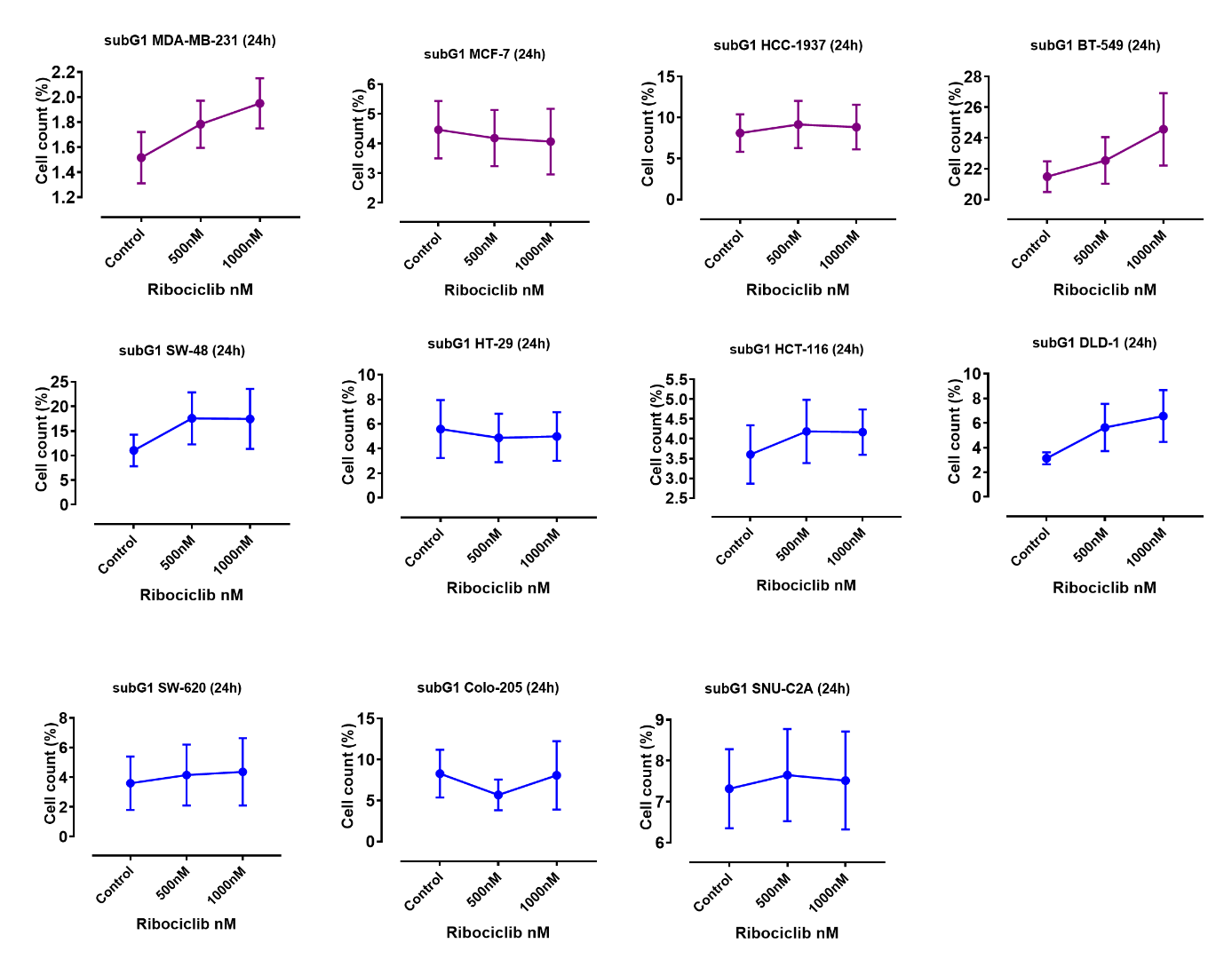


**Suppl. Figure 1 Sub-G1 events under CDK4/6 inhibition.** The figure illustrates the measured Sub-G1 events upon treatment with ribociclib in investigated cell lines (n = 3, not significant. ANOVA)


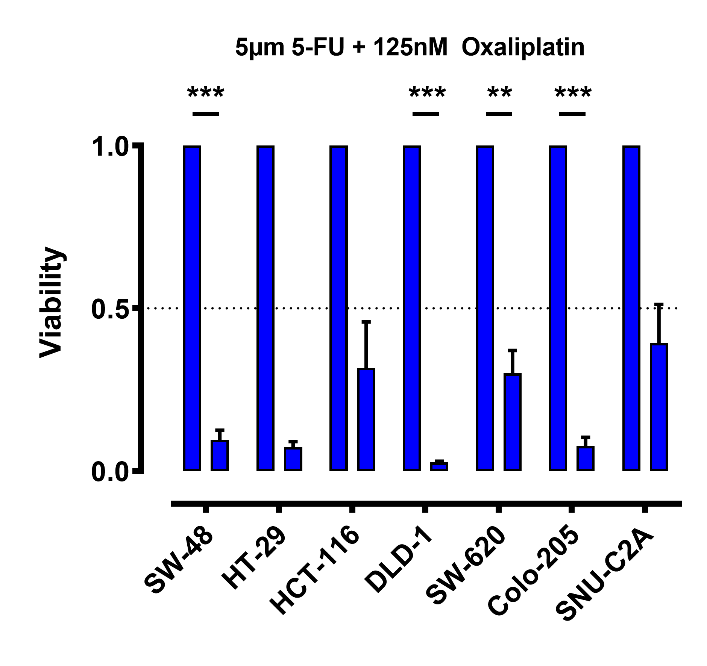


**Suppl. Figure 2 Sensitivity to FOLFOX of investigated CRC cell lines.** The figure illustrates the sensitivity of the investigated CRC cell lines to FOLFOX (n = 3, * p < 0.05, ** p < 0.01, *** p < 0.001, t- test or Mann-Whitney U test)


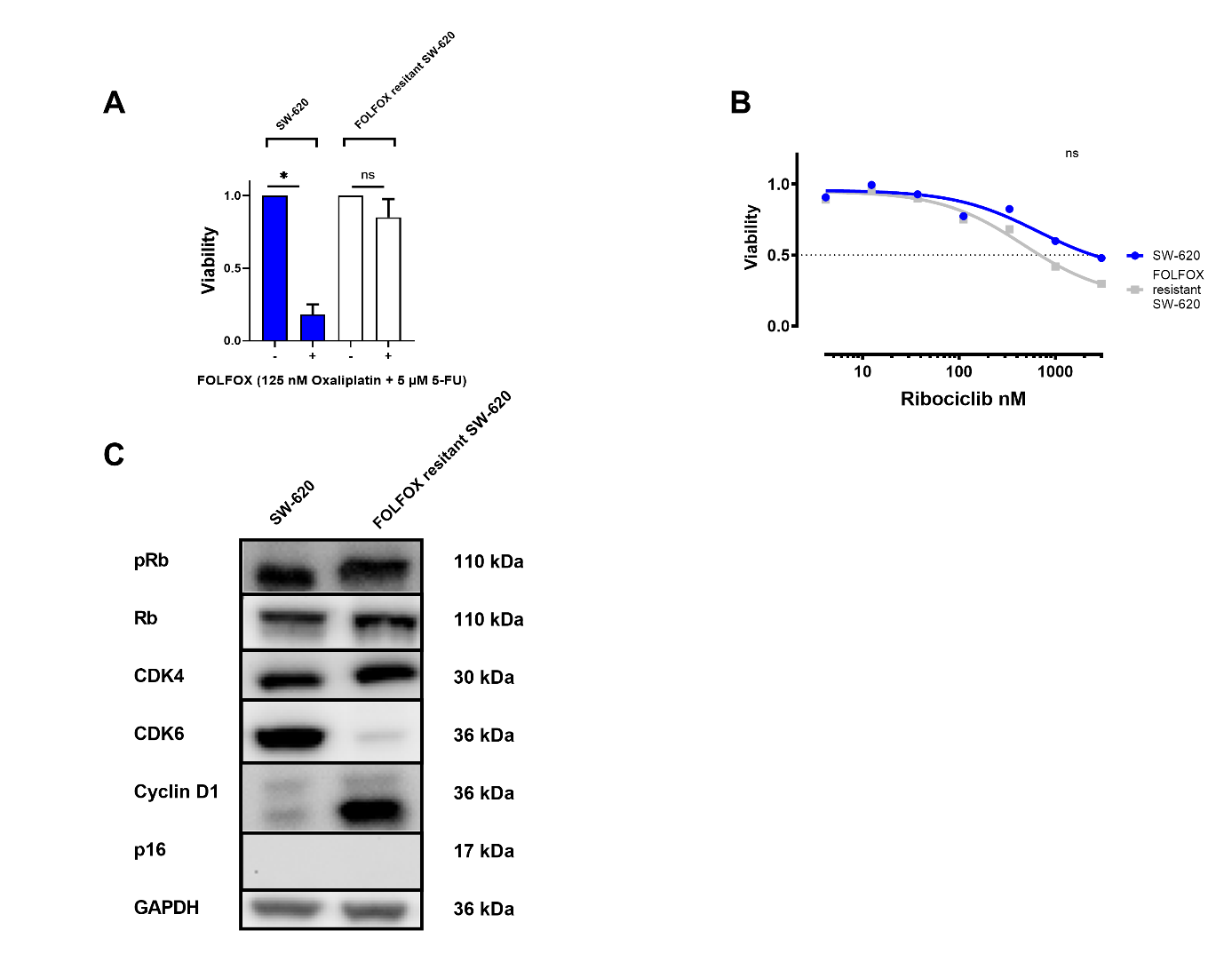


**Suppl. Figure 3 Antiproliferative effects of pharmacological CDK4/6 inhibition in a FOLFOX-resistant CRC cell line.** (A) Proliferation assay of sensitive SW-620 or FOLFOX-resistant SW-620 cells in the presence of 5 µM 5-FU and 125 nM oxaliplatin (FOLFOX) (n = 3; * p < 0.05, t.test). (B) The antiproliferative efficacy of ribociclib therapy in sensitive or FOLFOX-resistant SW-620 cells is illustrated (n = 3; ns = not significant; ANOVA). (C) The Western blot shows the protein expression profile of sensitive and resistant SW-620 cells. Representative blots of 3 independent experiments are shown


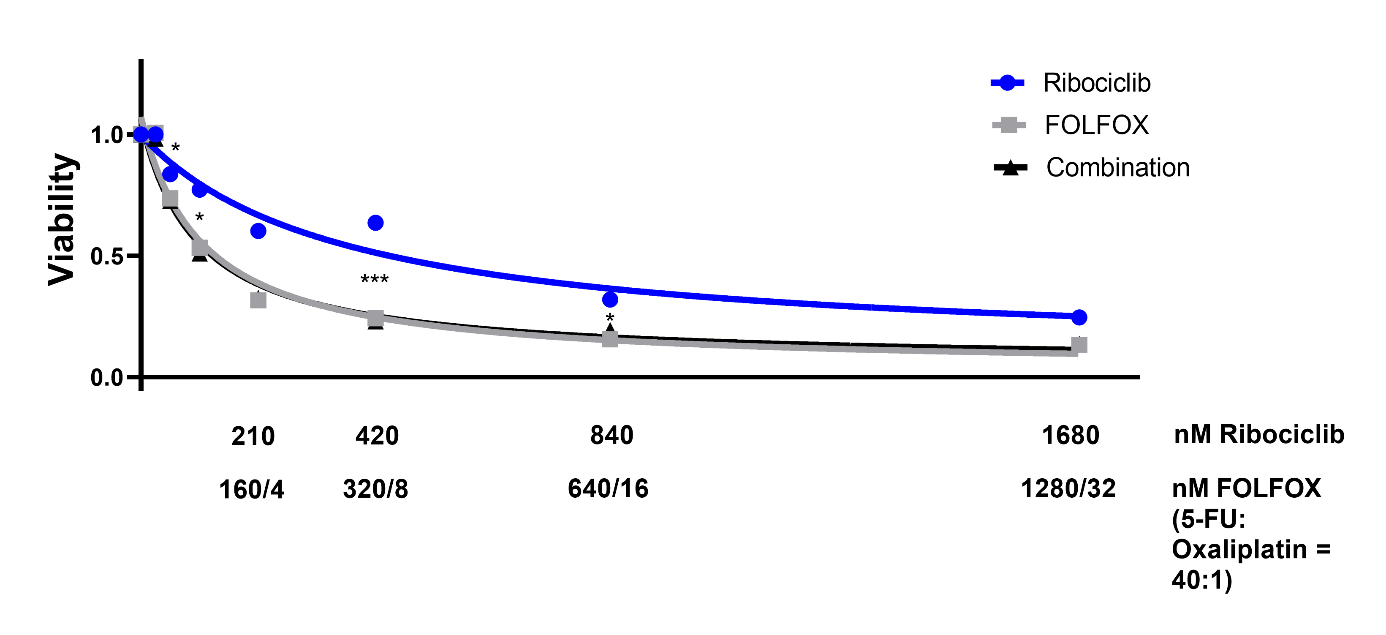


**Suppl. Figure 4 Synergism analysis of a cytostatic chemotherapy with CDK4/6 inhibition by ribociclib.** The figure shows a proliferation assay under FOLFOX, ribociclib, or FOLFOX + ribociclib in a ribociclib-sensitive cell line (Colo-205) (n = 3, * p < 0.05, ** p < 0.01, *** p < 0.001, ANOVA)


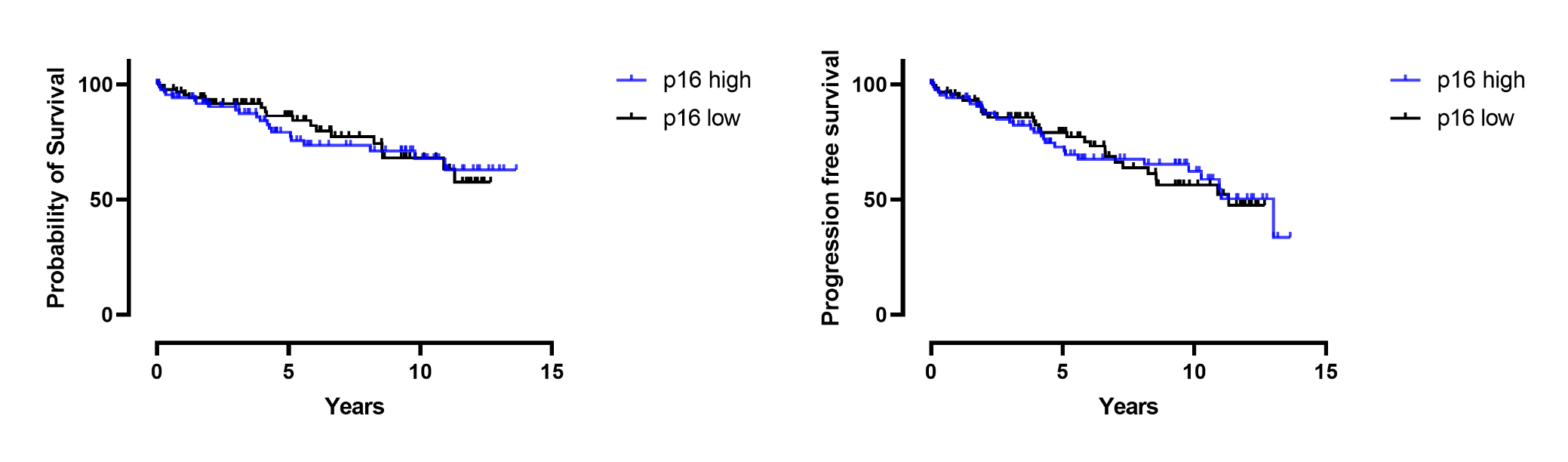


**Suppl. Figure 5 Survival analysis of a CRC cohort in UICC stage II.** In this analysis, tumor tissue from n=185 CRC patients was examined. The p16 protein expression of all patients was determined using immunohistochemistry. The median was used as the threshold for distinguishing between p16low and p16high (n=185; not significant; log-rank test)


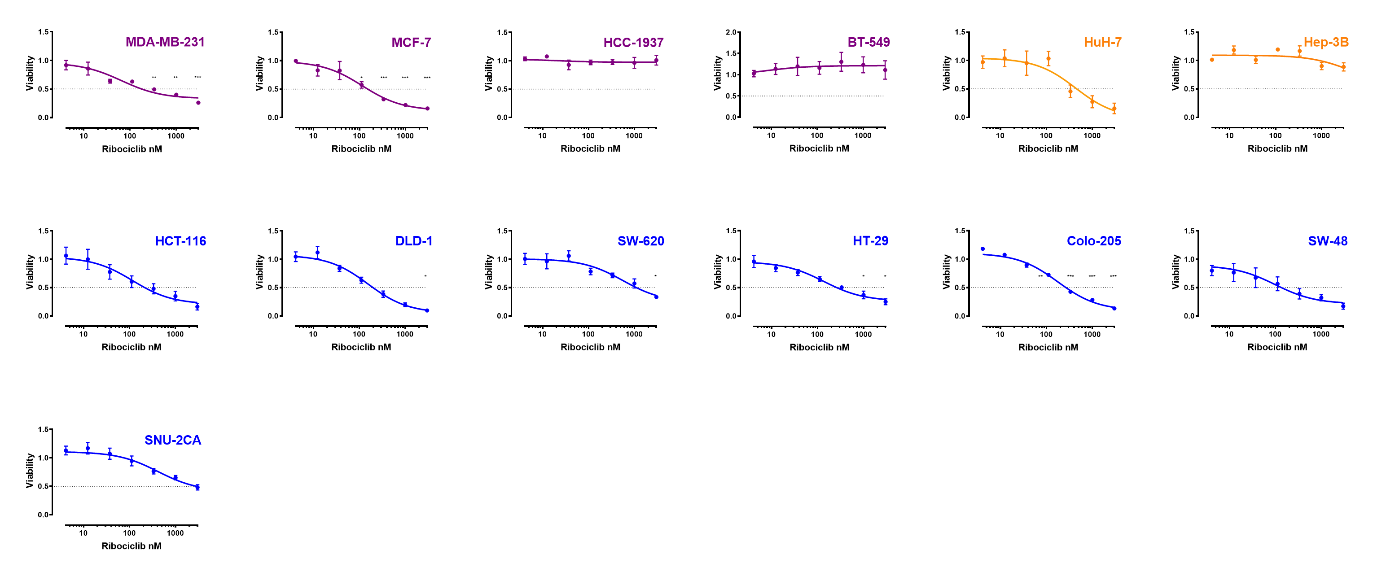


**Suppl. Figure 6 Sensitivity to ribociclib of investigated cell lines.** The figure illustrates the sensitivity of the investigated BC cell lines (purple), HCC cell lines (yellow), and CRC cell lines (blue) to ribociclib (n = 3, * p < 0.05, ** p < 0.01, *** p < 0.001, ANOVA or Kruskal-Wallis)


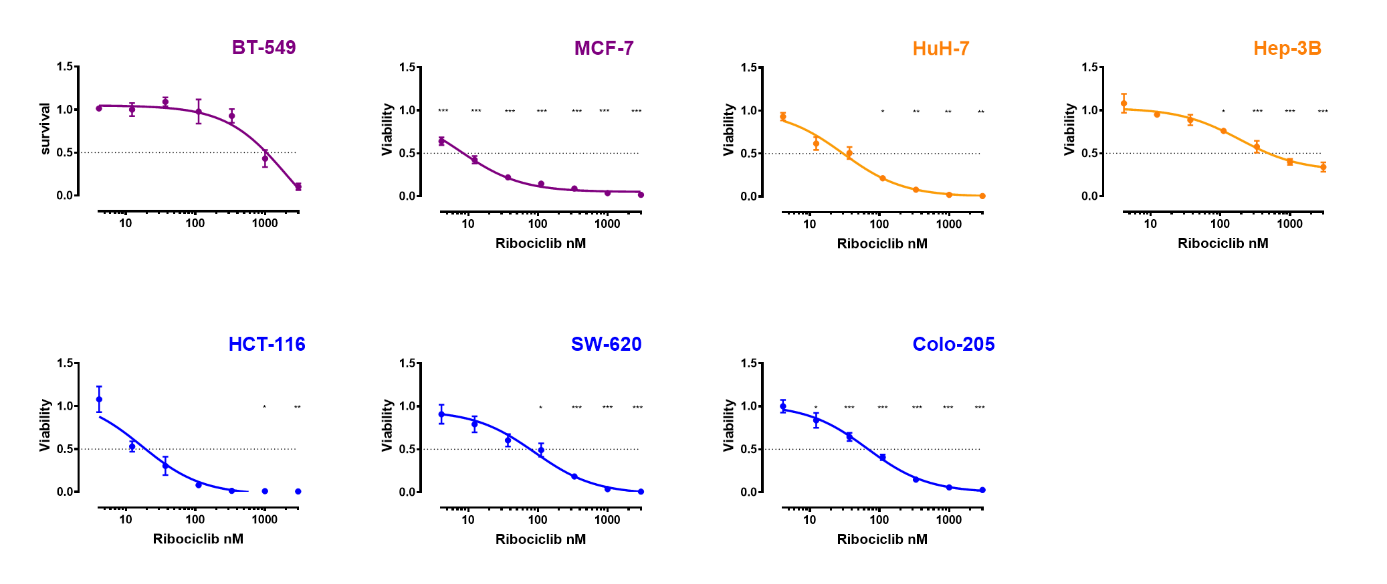


**Suppl. Figure 7 Sensitivity to abemaciclib of investigated cell lines.** The figure illustrates the sensitivity of the investigated BC cell lines (purple), HCC cell lines (yellow), and CRC cell lines (blue) to abemaciclib (n = 3, * p < 0.05, ** p < 0.01, *** p < 0.001, ANOVA or Kruskal-Wallis)


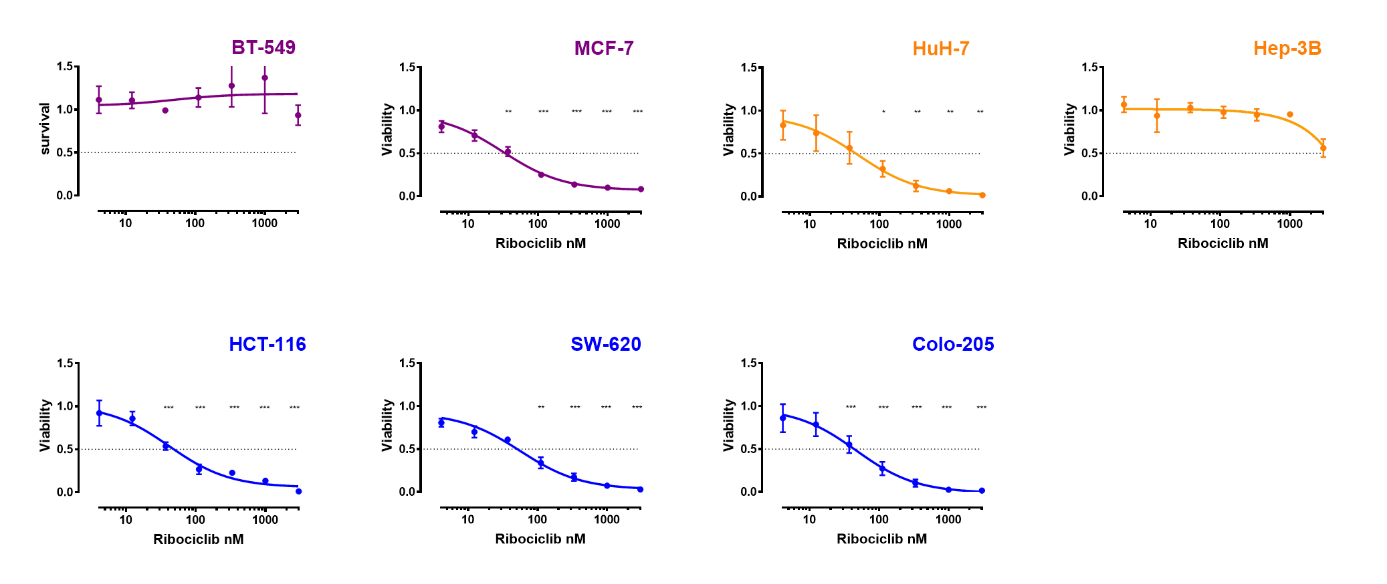


**Suppl. Figure 8 Sensitivity to palbociclib of investigated cell lines.** The figure illustrates the sensitivity of the investigated BC cell lines (purple), HCC cell lines (yellow), and CRC cell lines (blue) to palbociclib (n = 3, * p < 0.05, ** p < 0.01, *** p < 0.001, ANOVA or Kruskal-Wallis)
